# Carbon Materials Derived from Waste Streams: From Processing Pathways to Structure–Property–Function Relationships

**DOI:** 10.3390/ma19102146

**Published:** 2026-05-20

**Authors:** Sharif H. Zein

**Affiliations:** 1 Faculty of Engineering, Sohar University, Sohar 311, Oman; szein@su.edu.om or s.h.zein@hull.ac.uk; 2 Faculty of Science and Engineering, School of Engineering, University of Hull, Hull HU6 7RX, UK

**Keywords:** waste-derived carbon materials, pyrolysis, hydrothermal carbonisation, hierarchical porous carbon, structure–property relationships, electrochemical energy storage, waste valorisation policy

## Abstract

**Highlights:**

**What are the main findings?**

- Waste biomass, plastics, and sludge yield functional carbons via pyrolysis and HTC.
- Feedstock lignin, cellulose, and ash content governs pore architecture and surface chemistry.
- Heteroatom doping and defect engineering improve conductivity and electrocatalytic activity.

**What are the implications of the main findings?**

- Waste streams are viable precursors that reduce dependence on fossil-derived activated carbon.
- Processing route selection is the primary control over carbon structure for any given feedstock.
- Standardised protocols and ML optimisation are required to bring advances to industrial scale.

**Abstract:**

The accelerating generation of waste streams is observed globally. Spanning lignocellulosic biomass, plastic waste, sewage sludge, and industrial residues, this review presents both an urgent management challenge and a compelling materials opportunity. Carbon materials derived from these waste streams offer a sustainable route to functional carbons applicable in electrochemical energy storage, adsorption, heterogeneous catalysis, and high-temperature applications. Yet their rational design remains constrained by incomplete understanding of the relationships between feedstock composition, processing pathway, structural characteristics, and functional performance. This review provides an integrated analysis of waste-derived carbon materials from processing pathways to structure–property–function relationships. The principal feedstock categories are examined for their compositional characteristics and implications for carbon yield and structure. Five primary processing routes are assessed. The five routes examined are pyrolysis, hydrothermal carbonisation, physical and chemical activation, and microwave-assisted processing. They are assessed comparatively with emphasis on structural outcomes and governing parameters. The resulting structural characteristics are discussed. These are morphology, hierarchical pore architecture, surface chemistry, heteroatom doping, and crystallinity. They are discussed alongside their characterisation methods and known limitations as performance predictors. Structure–property relationships are examined quantitatively. Heteroatom-doped hierarchical porous carbons achieve 612 F/g specific capacitance. Turbostratic hard carbons deliver 450 mAh/g sodium storage with over 90% retention. Hierarchical porous carbons demonstrate CO_2_ uptake of 5.0 mmol/g and dye adsorption exceeding 9000 mg/g under optimised laboratory conditions; these values reflect individual studies and are not directly comparable across systems. Biomass-derived sulfonated carbon catalysts sustain biodiesel yields above 90% over multiple cycles. Challenges of feedstock variability, process scalability, environmental compliance, and economic feasibility are addressed, and machine learning-guided design, standardised characterisation methodology, and circular economy policy frameworks are identified as key enablers for translating laboratory performance into industrial reality.

## 1. Introduction

The scale of global waste generation has become one of the defining material challenges of this century. Municipal solid waste production is projected to rise substantially by mid-century, driven principally by population growth, accelerating urbanisation, and the expansion of consumption-intensive economies [1,2]. Plastic waste presents a particular difficulty. Global production now surpasses 390 million tonnes per year, with effective recycling rates below 10%, and a substantial proportion of this material accumulates in terrestrial and aquatic environments rather than re-entering productive use [3]. Agricultural and forestry residues add a further dimension to this picture. Of the approximately 170 gigatonnes of lignocellulosic biomass generated annually worldwide, only around 5% is currently directed toward food or non-food applications, leaving an enormous resource base largely untapped [4]. Conventional approaches to managing these waste streams, principally landfilling and incineration, are well-documented in their environmental costs, from methane and carbon dioxide emissions to soil and groundwater contamination. Neither offers a route toward material recovery [2,5].

Against this background, there has been sustained and growing interest in valorisation strategies that treat waste not as a disposal problem but as a feedstock for useful materials. Carbon-based materials occupy a particularly important position within this broader effort. Biochar, activated carbon, hydrochar, and hard carbon can all be produced from waste precursors, and each offers structural and chemical characteristics that can be tuned, within limits, to meet specific functional requirements [6,7]. These materials have attracted serious attention across adsorption, environmental remediation, heterogeneous catalysis, and electrochemical energy storage. In these fields, demand for low-cost, high-performance carbon materials continues to outpace supply from conventional fossil-derived sources [8,9,10].

The principal thermochemical conversion routes for these materials are pyrolysis, hydrothermal carbonisation, and microwave-assisted carbonisation. They differ considerably in their operating conditions, energy demands, and the types of feedstock they can accommodate [11,12,13,14,15]. What they share is sensitivity to processing parameters: temperature, heating rate, residence time, and the presence or absence of activating agents all exert measurable influence over the pore architecture, surface chemistry, and degree of graphitic ordering in the resulting carbon [16]. The relationship between these processing variables and the structural characteristics of the product is therefore not incidental but central to the rational design of waste-derived carbons for specific end uses.

This brings into focus a principle that runs throughout materials science: structure, properties, and function are interdependent. For waste-derived carbons, porosity and surface area govern adsorption capacity; graphitic ordering and defect density determine electrical conductivity and electrochemical behaviour; and the nature and density of surface functional groups shape catalytic activity and chemical selectivity [16,17]. Establishing reliable structure–property–function relationships for these materials is, however, considerably more difficult than for carbons produced from well-defined synthetic precursors, precisely because waste feedstocks are inherently heterogeneous in composition and variable between sources and seasons [18,19].

A substantial body of literature has accumulated on waste-derived carbon materials over the past decade, and several reviews have addressed particular aspects of the field. Biochar production from food waste and its environmental applications have been reviewed in previous studies. [8,20] Wood-based biochar has been examined specifically for the removal of potentially toxic elements from water [21]; The conversion of PET waste into nanoporous carbon for contaminant adsorption has been addressed [22]; and thermochemical processing of agri-food and lignocellulosic biomass wastes has been surveyed more broadly [7,13]. These contributions are valuable, but they tend to focus on a single feedstock class or a specific application domain. What has been less systematically addressed is the connective tissue between these areas. Specifically, the connective tissue is how processing route selection, feedstock composition, structural development, and functional performance relate to one another across the full range of relevant waste sources.

This review addresses that gap by analysing waste-derived carbon materials across the full processing chain, from feedstock composition through thermochemical conversion to functional performance. Unlike prior reviews focused on individual feedstock classes or single applications, this work synthesises structure–property–function relationships across biomass, plastic, sludge, and industrial waste streams within a single comparative framework. It covers the principal classes of waste feedstock available for carbon material production, the thermochemical conversion routes through which they are processed, the structural characteristics that result, and the functional consequences of those characteristics in adsorption, catalysis, and energy storage applications. Scalability, reproducibility, and the practical constraints on moving from laboratory demonstration to industrial implementation are also considered. The aim throughout is not to catalogue the literature but to draw out the material relationships that govern performance and to identify where current understanding remains incomplete. A comparative analysis of a single feedstock subjected to different processing routes is provided in Section 3 to illustrate how processing conditions, rather than feedstock alone, determine structural and functional outcomes.

The cited literature spans studies of varying experimental rigour and characterisation depth. Where studies report conflicting results, this review identifies the likely source of disagreement, typically differences in feedstock composition, activation conditions, or characterisation protocol. Performance data cited throughout reflect optimised laboratory conditions and represent upper bounds on achievable performance rather than typical outcomes.

## 2. Waste Feedstocks for Carbon Material Production

**Figure 1:**
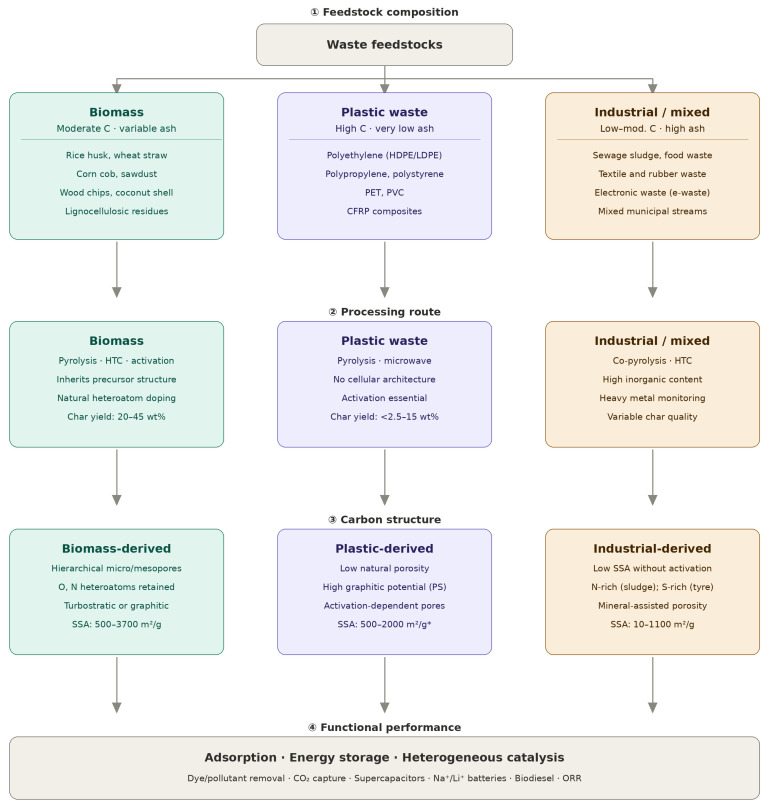
Structure–property–function framework for waste-derived carbon material production. Feedstock compositional characteristics (① lignin, cellulose, and ash content) determine processing behaviour (②), which controls carbon structural properties (③ porosity, surface chemistry, graphitic ordering, and heteroatom content) and ultimately functional performance (④). SSA = specific surface area. * After chemical activation (KOH, H_3_PO_4_, or ZnCl_2_). Green represents biomass, purple represents plastic waste, and amber represents industrial/mixed feedstock categories throughout the figure.

The nature of the waste feedstock is not merely a starting point in the production of carbon materials. It is a primary determinant of what is structurally and chemically achievable in the final product. The intrinsic composition of any given waste material, including its carbon content, volatile matter fraction, ash loading, and moisture level, governs thermal decomposition behaviour, carbon yield, pore formation, and surface chemistry throughout thermochemical processing [23,24,25]. Waste materials suitable for carbon production fall broadly into three categories: biomass-derived waste, plastic and polymeric waste, and mixed or industrial waste streams. Each exhibits distinct carbonisation characteristics that place different demands on processing strategy and impose different constraints on the properties of the resulting carbon. A consolidated overview of representative feedstocks across these categories, including their typical compositional ranges and principal advantages and limitations, is presented in Table 1. Figure 1 provides a conceptual overview of these three feedstock categories and their principal processing characteristics.

**Table 1:** Representative waste feedstocks for carbon material production: compositional characteristics, principal advantages, and key limitations.

Feedstock Category	Representative Examples	Typical Carbon Content (wt%)	Typical Ash Content (wt%)	Dominant Structural Component	Key Consideration	Key Limitation	Key References
Biomass—Agricultural	Rice husk	35–40	15.7–21	Cellulose + silica	Advantage: Abundant; distinctive silica-assisted pore formation	High ash blocks micropores	[26,27]
	Coconut shell	53–64	Low (<5)	Lignin (41–45%)	Advantage: Dense framework; high mechanical stability; high carbon yield	Limited regional availability	[28,29]
	Wheat straw	38–42	Moderate	Cellulose (39–41%)	Advantage: Abundant agricultural residue; microporous carbon	Variable composition; Ca/K catalytic effects	[30,31]
	Corn cob/Barley husk	42–45	Low–moderate	Cellulose (52–79%)	Advantage: High cellulose; good activated carbon precursor	Seasonal availability	[7,25]
Biomass—Forestry	Sawdust/Wood chips	45–55	Low	Lignocellulosic (balanced)	Advantage: Relatively uniform structure; scalable supply.	Moisture sensitivity	[32,33]
Plastic— Polyolefins	Polyethylene (PE)	80–90	Very low	Aliphatic hydrocarbon	Advantage: High theoretical carbon content; low ash.	Minimal solid char without activation (<2.5 wt%); no natural porosity	[34,35]
	Polypropylene (PP)	80–90	Very low	Aliphatic hydrocarbon	Advantage: Similar to PE; abundant waste stream	Very low char yield (~1.6 wt%); activation required	[34,36]
Plastic— Aromatic	PET	60–70	Low	Aromatic polyester	Advantage: Graphitic development potential	Reduces liquid yield in mixed streams	[35,37]
	Polystyrene (PS)	85–92	Very low	Aromatic hydrocarbon	Advantage: High aromatic content; improved conductivity potential	Predominantly depolymerises to monomer	[35,38]
Plastic— Halogenated	PVC	35–55	Low–moderate	Chlorinated polymer	Advantage: Higher char yield than polyolefins (6–9 wt%)	HCl emissions; requires gas treatment; corrosion risk	[34,39]
Industrial	Sewage sludge	20–30	High (>30)	Organic matter + minerals	Advantage: Large-scale availability; nitrogen-rich carbon	Low surface area (10–150 m^2^/g); heavy metal contamination	[40,41]
	Textile waste	40–60	Moderate	Cellulosic/synthetic fibre	Advantage: Fibre morphology retained; hierarchical porosity potential	Mixed composition variability	[7,42]
	Rubber/Tyre waste	60–75	Moderate	Aromatic polymer + carbon black	Advantage: Conductive framework; high carbon content	Sulphur content; additive contamination	[42,43]
	Electronic waste (PCB)	52–74 *	High	Thermoset polymer + glass fibre	Advantage: High recoverable carbon (up to 25 wt%); circular economy potential	Brominated flame retardants; toxic metals	[34,44]
	Composite waste (CFRP)	Variable	Variable	Carbon fibre + polymer matrix	Advantage: Carbon fibre recovery viable at 700 °C	Separation complexity; cost of pre-treatment	[45,46]

Note: Carbon yield, BET surface area, and application performance vary considerably with processing route and activation conditions and are summarised comparatively in Table 2. * Carbon content varies significantly depending on PCB type and composition.

**Table 2:** Consolidated comparison of principal thermochemical processing routes for waste-derived carbon material production.

Processing Route	Operating Conditions	Primary Carbon Product	Typical Surface Area Achievable (m^2^/g)	Dominant Pore Type	Key Considerations	Key Limitation	Key References
Slow pyrolysis	300–650 °C; inert atmosphere; <10 °C/min; long residence time	Biochar	100–800	Microporous	Advantage: High carbon yield; developed porous structure; stable aromatic framework	Limited surface area without activation; variable with feed-stock	[47,48,49]
Fast pyrolysis	400–700 °C; inert atmosphere; >100 °C/s; residence time < 2 s	Low-porosity char co-product	7–50	Poorly developed	Advantage: Maximises liquid bio-oil yield as primary product	Very low char surface area; minimal porosity without activation	[48]
Hydrothermal carbonisation (HTC)	180–260 °C; subcritical water; autogenous pressure; no pre-drying	Hydrochar	<50 as-produced; >2400 after activation	Micro-mesoporous after activation	Advantage: Processes wet feedstocks directly; rich oxygen functional groups; mesoporous after activation	Low as-produced surface area; pressure-resistant reactor required	[5,50,51]
Physical activation—steam	800–1100 °C; steam; two-stage process	Activated carbon	400–1200	Microporous with mesopores	Advantage: Environmentally benign; no chemical recovery; tubular pore structure	Lower sur-face area than KOH; high energy input; long processing time	[52,53,54]
Physical activation—CO_2_	800–1100 °C; CO_2_; Boudouard reaction	Activated carbon	250–900	Microporous	Advantage: Clean process; well-defined micropores; controllable	Lower surface area than steam; high	[54,55]
Chemical activation—KOH	400–800 °C; KOH impregnation; K intercalation	Highly microporous activated carbon	Up to 3700	Predominantly microporous	Advantage: Highest achievable surface areas; single step; lower temperature than physical	Corrosive reagent; acid washing required; chemical wastewater	[52,56,57]
Chemical activation—H_3_PO_4_	~500 °C; phosphate ester cross-linking	Mesoporous activated carbon	330–1720	Micro-mesoporous	Advantage: Lower temperature than KOH; mesopore development; lower toxicity than ZnCl_2_	Lower surface area than KOH; sintering risk above 500 °C	[57,58,59]
Chemical activation—ZnCl_2_	500–800 °C; dehydrating agent	Tunable micro-mesoporous carbon	900–2300	Tunable micro-mesoporous	Advantage: Adjustable pore architecture; higher carbon yield than KOH	Advantage: Adjustable pore architecture; higher carbon yield than KOH	[57,60]
Microwave-assisted processing	500–1200 °C equivalent; internal volumetric heating; 300–1000 W	Biochar/activated carbon	Up to 2996	Ultra-microporous to hierarchical	Advantage: Up to 80% processing time reduction; higher graphitic character; unique nanostructures possible	Hot spot formation; dielectric variability; limited industrial scale	[61,62,63]

Note: Surface area values represent reported maxima under optimised conditions for each route and are not directly comparable across feedstocks or experimental systems.

### 2.1. Biomass and Agricultural Waste

Biomass-derived waste materials occupy a central position in the production of waste-derived carbons, principally because of their natural abundance, the diversity of available sources, and the structural complexity that their lignocellulosic composition imparts to the resulting carbon frameworks. Agricultural residues, forestry by-products, and food-processing wastes all fall within this category. Their suitability as carbon precursors depends on the relative proportions of three major structural constituents: cellulose, hemicellulose, and lignin [7,23,25,64].

Cellulose is a linear polysaccharide with an elemental composition of approximately 44.4% C, 49.4% O, and 6.2% H by weight [7]. Its crystalline structure renders it moderately thermally stable, with decomposition occurring primarily between 280 and 400 °C, producing a single sharp mass loss peak in thermogravimetric analysis [65,66,67]. Hemicellulose, by contrast, is amorphous and branched, and accordingly decomposes at lower temperatures, typically between 180 and 315 °C [67,68,69]. Lignin is a three-dimensional aromatic polymer with approximately 62% carbon content, markedly higher than either cellulose or hemicellulose [7,25]. Its complex network of phenylpropane units confers exceptional thermal stability, with degradation occurring across the wide range of approximately 160 to 900 °C [30,67,69,70]. These three components are not thermally independent. Interactions between them during pyrolysis shift decomposition temperatures and alter char yields relative to what isolated components would produce [65,68].

The practical consequence of this compositional framework for carbon material design is well established. Feedstocks with high lignin content produce higher solid carbon yields because lignin decomposes slowly over a wide temperature range, contributing disproportionately to char formation [29,30,71]. High lignin content also promotes the development of macroporous carbon frameworks [7,25]. Cellulose-rich feedstocks, on the other hand, tend to produce predominantly microporous structures, though at the cost of lower carbon yields [7,25,72]. Ash content introduces a further complication: high mineral loading blocks micropores, creates inactive surface sites, and reduces the mechanical integrity of the resulting adsorbent [29,73]. Biomass samples with carbon content above 40%, ash below 10%, and moisture below 30% are generally regarded as well-suited precursors for activated carbon production [25].

Quantitative data from component-level studies confirm these trends. Under pyrolysis without activation, cellulose produces a microporous char with BET surface area up to 394 m^2^/g, whilst lignin and xylan yield chars with surface areas below 10 m^2^/g [74]. Char yield follows the opposite pattern: lignin produces approximately twice the char yield of cellulose or hemicellulose, reflecting its thermal stability across a broad decomposition range of 160–900 °C [75]. Upon KOH activation, surface area rankings shift considerably: xylan-derived carbon reaches 925 m^2^/g, cellulose 678 m^2^/g, and lignin 513 m^2^/g [74]. Under H_3_PO_4_ activation, lignin-derived carbons achieve higher micropore volumes than cellulose at equivalent temperatures, whilst cellulose yields higher mesopore volumes above 350 °C [76]. Feedstock lignocellulosic composition therefore determines both carbon yield and pore architecture, and activation agent selection further governs the structural outcome.

Several agricultural residues have been studied extensively as carbon precursors precisely because they combine favourable composition with large-scale availability. Rice husk is distinguished by its exceptionally high silica content, with ash levels of approximately 15.7 to 21 wt%. This influences pore architecture during carbonisation and can trap free radicals within a molten amorphous silica matrix at high temperatures [26,31]. This silica has been shown to participate in char radical formation in a manner distinct from other agricultural residues [31]. Coconut shell, by contrast, is characterised by a high lignin content of 41 to 45 wt% and a dense cross-linked lignocellulosic structure that produces carbon materials with high mechanical stability and dense carbon frameworks [28,29]. This structural compactness, arising from stronger inter-component cross-linking than is typical of most agricultural biomass, makes coconut shell-derived carbon particularly suited to demanding applications such as electrocatalyst supports and high-performance adsorbents [28,77]. Sawdust and wood-derived biomass exhibit relatively uniform composition compared with agricultural residues, which translates into more predictable carbonisation behaviour and the potential for well-ordered carbon structures during processing [32,33].

Ash and inorganic components are not, however, uniformly detrimental. Potassium, one of the most abundant alkali metals in biomass ash, catalyses char-forming reactions and secondary cracking of volatiles during pyrolysis, increasing char yield by approximately 5–7 wt% compared with demineralised feedstocks [78]. Removal of mineral species from sugar cane bagasse prior to pyrolysis opened channel networks and increased meso- and macroporosity, confirming that metal ions previously blocking pore access were partially eliminated during demineralisation [79]. The net effect of ash on carbon structure therefore depends on mineral speciation: alkali metals promote char yield through secondary charring reactions, whilst inert or fusible minerals reduce surface area through pore blockage.

Chemical activation markedly extends the performance of biomass-derived carbons beyond what direct pyrolysis achieves. Potassium hydroxide activation operates through redox reactions between KOH and the carbon framework at temperatures above 700 °C, consuming carbon atoms and generating a highly microporous network. Surface areas exceeding 2000 m^2^/g have been reported for KOH-activated coconut shell carbons under optimised conditions [80,81,82]. Phosphoric acid activation operates at lower temperatures, around 500 °C, through a dehydrating mechanism that forms phosphate ester cross-links within the biomass structure and promotes mesopore development [8,83,84]. Zinc chloride activation offers a tunable route to both micro- and mesoporous structures depending on impregnation ratio and temperature, making it particularly versatile for applications where hierarchical pore architecture is desirable [33,83,85].

A persistent challenge with biomass feedstocks is compositional variability. Lignin concentration, moisture content, and mineral composition fluctuate with plant species, cultivation conditions, season, and geography [27,30,86]. These variations translate directly into inconsistency in carbon yield, pore structure, and surface chemistry, and represent a significant obstacle to reproducible large-scale production. Careful feedstock characterisation and standardised pre-treatment procedures are therefore not optional refinements but necessary preconditions for reliable materials performance. This variability also affects the reliability of performance metrics reported in the literature. BET surface area, adsorption capacity, and capacitance values obtained from a single well-characterised batch cannot be assumed to represent the range of outcomes achievable from the same nominal feedstock under the same processing conditions. Reported performance maxima should therefore be interpreted as upper bounds rather than typical values.

### 2.2. Plastic and Polymeric Waste

Global plastic production now surpasses 390 million tonnes per year, with effective recycling rates below 10% [3]. The resulting accumulation of polymeric waste in both terrestrial and aquatic environments has driven sustained interest in thermochemical conversion as an alternative to disposal. Unlike lignocellulosic biomass, plastic feedstocks do not possess inherent microstructural porosity, and their carbonisation behaviour is governed primarily by polymer backbone chemistry rather than the staged decomposition of distinct biopolymer components.

Polyethylene and polypropylene are the most abundant polymeric waste streams. Both undergo thermal decomposition through random-chain scission, generating predominantly gaseous and liquid hydrocarbon products. Solid carbon residue is typically below 2.5 wt% at standard pyrolysis temperatures [34,35,36]. Their aliphatic structures offer limited scope for graphitic domain formation, and activation is invariably required to develop any useful pore architecture in carbon materials derived from these polymers [87]. Polystyrene presents a rather different picture. Its aromatic backbone promotes depolymerisation back to styrene monomer under thermal conditions and supports the formation of carbon materials with improved electrical conductivity due to the greater tendency for aromatic cross-linking during carbonisation [35,38]. Carbon yield is a practical viability criterion that receives insufficient attention relative to structural properties in the literature. For polyolefin feedstocks, char yields below 2.5 wt% mean that over 97% of input material converts to oil and gas, requiring large feedstock volumes to produce meaningful carbon quantities [34,35,36]. Biomass feedstocks yield substantially higher char fractions, making them considerably more efficient as carbon precursors at scale. Waste plastic-derived carbons have demonstrated specific surface areas exceeding 3000 m^2^/g and promising electrochemical performance in supercapacitors and batteries [88].

Polyethylene terephthalate contains aromatic ester linkages that result in more complex decomposition behaviour than simple polyolefins. Increasing PET content in mixed pyrolysis feeds has been shown to reduce liquid yields substantially while increasing gas formation and solid residue [35,37]. Its aromatic character confers some graphitic development potential, particularly under high-temperature treatment. Polyvinyl chloride is the most problematic of the common plastic feedstocks from a processing perspective. Thermal decomposition occurs in two stages: dehydrochlorination between 200 and 360 °C, releasing substantial quantities of hydrogen chloride gas, followed by backbone degradation between 360 and 550 °C producing aromatic organic substances and additional chlorinated compounds [35,39]. Char yields from PVC are higher than from polyolefins, at approximately 6 to 9 wt%, due to cross-linking reactions following dehydrochlorination [34]. The corrosive and environmentally hazardous nature of HCl emissions necessitates careful gas treatment and, in mixed feedstream scenarios, dechlorination strategies [39,89].

Mixed plastic waste streams present additional complexity: average solid carbon yields of approximately 10 to 20 wt% have been reported, with product distributions highly dependent on waste composition [90,91]. Co-pyrolysis of plastic waste with biomass addresses both waste streams simultaneously. Interactions between biomass and plastic components reduce oxygenated content in pyrolysis oils and can improve overall carbon yield relative to either feedstock processed alone [92,93].

The formation of toxic by-products during plastic pyrolysis, including volatile organic compounds, polycyclic aromatic hydrocarbons, and halogenated species, is a serious practical concern that cannot be minimised by feedstock selection alone [39,44,94]. Effective gas treatment systems, controlled reaction atmospheres, and consistent feedstock sorting are prerequisites for environmentally responsible production of carbon materials from plastic waste at any meaningful scale.

Feedstock pre-screening for chlorine and bromine content is the most effective first-line measure for reducing halogenated emission risks during plastic and e-waste pyrolysis [39,44]. Where halogenated feedstocks cannot be avoided, staged condensation and alkaline scrubbing systems have been demonstrated to achieve high removal efficiencies for acid gases and halogenated species from pyrolysis off-gas [94]. The justification for processing halogenated plastic and e-waste streams requires weighing landfill or incineration impacts against pyrolysis emission hazards and the value of the recovered carbon product [39,44]. Where effective gas treatment is in place and feedstock chlorine content is controlled, pyrolysis can deliver a net environmental benefit, particularly when the carbon product displaces fossil-derived activated carbon [94].

### 2.3. Industrial and Mixed Waste Feedstocks

Industrial and mixed waste streams broaden the feedstock base considerably but introduce a degree of compositional heterogeneity that creates distinct challenges for material reproducibility and environmental safety. This category encompasses sewage sludge, textile residues, rubber waste, electronic waste, and composite materials, each presenting a different combination of organic carbon, inorganic mineral phases, and potentially hazardous constituents.

Sewage sludge is one of the most extensively studied industrial feedstocks due to the sheer volume generated by municipal wastewater treatment globally and the dual benefit of simultaneous waste volume reduction and carbon material production. Sludge contains organic matter amenable to carbon formation alongside significant quantities of inorganic minerals and heavy metals [41,95,96]. Direct pyrolysis of sewage sludge typically yields biochars with surface areas of 10 to 150 m^2^/g, substantially lower than plant-derived biochars. The high inorganic content blocks pore development and can cause structural collapse at elevated temperatures [40,97]. Co-pyrolysis with carbon-rich biomass materials such as hazelnut shells or wheat straw has been shown to improve pore structure markedly, increasing surface area and reducing heavy metal concentrations in the resulting biochar [95,98,99]. Chemical activation, particularly with KOH, can bring sludge-derived activated carbon surface areas to approximately 1000 m^2^/g, approaching but generally remaining below commercial activated carbon benchmarks [40,100,101]. A critical consideration is heavy metal fate. Pyrolysis immobilises metals by converting weakly bound forms to stable mineral phases, but leaching risk must be evaluated carefully, particularly where co-pyrolysis or activation conditions alter metal speciation [99,102,103].

Textile waste, particularly cotton and polyester blends, offers a fibre-derived carbon precursor. The original fibre morphology can be partially retained during controlled carbonisation, yielding carbon materials with inherent hierarchical porosity [7,42,104]. Char from textile waste has been identified as a candidate raw material for activated carbon production for adsorption applications, though mixed synthetic-natural compositions introduce variability into the carbonisation pathway [42].

Waste rubber, principally from used tyres, combines high carbon content with an aromatic polymer structure that supports the formation of conductive carbon frameworks [42]. The sulphur incorporated during vulcanisation is retained in the pyrolysis char. Sulphur doping can enhance electrochemical activity, but it also limits the suitability of such carbons for applications requiring chemically clean surfaces [42,43]. Where pyrolysis conditions are optimised and catalytic additives employed, carbon nanotube-like structures with properties suitable for supercapacitor electrodes have been produced from tyre-derived feedstocks [42].

Electronic waste polymer fractions present the greatest complexity of any feedstock considered here. Polymer-rich printed circuit boards can yield up to 25 wt% recoverable carbon with carbon content reaching 52 to 74 wt% at high processing temperatures, substantially higher than individual commodity plastics processed under comparable conditions [34]. However, the co-presence of brominated flame retardants, lead, and other heavy metals generates hazardous by-products including hydrogen bromide and polybrominated compounds during thermal treatment [44,94]. Pre-treatment for metal and halogen removal is a prerequisite before thermal processing of e-waste fractions can be considered environmentally responsible [44]. Carbon fibre reinforced polymer composites represent a further niche within this category: pyrolysis at 700 °C in an inert atmosphere has been demonstrated to recover carbon fibres with acceptable mechanical and surface properties for use in secondary composite applications [45,46].

Across all industrial and mixed waste feedstocks, feedstock variability remains the dominant challenge. Variations in chemical composition and impurity loading translate directly into inconsistency in carbon material properties and introduce environmental risk at scale [18,41,42]. Systematic feedstock characterisation and process optimisation are therefore essential for ensuring that carbon materials from these sources achieve consistent structural and functional performance.

## 3. Processing Pathways for Carbon Material Production

**Figure 2:**
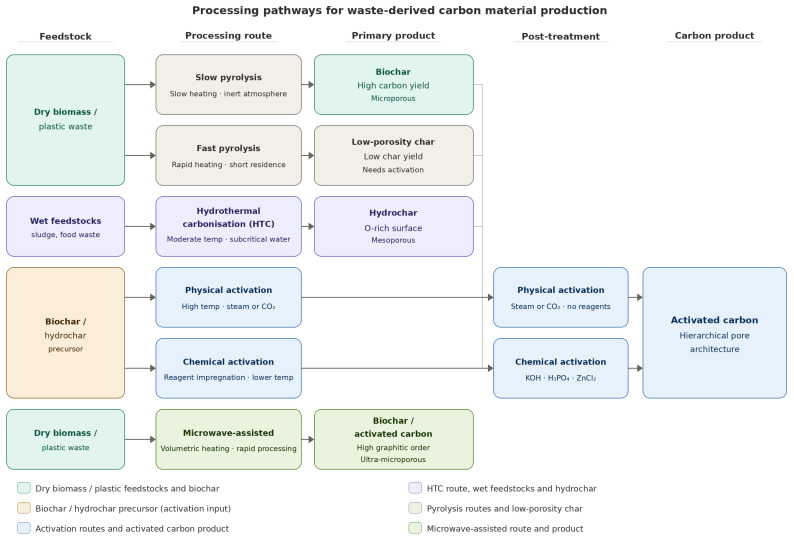
Processing pathways for waste-derived carbon material production. Primary carbonisation routes, namely slow pyrolysis, fast pyrolysis, and hydrothermal carbonisation (HTC), convert raw feedstocks into biochar or hydrochar, which serve as precursors for subsequent physical or chemical activation. Microwave-assisted processing represents an alternative primary route applicable to dry feedstocks. Colour coding identifies feedstock type, processing route, and product category as shown in the legend.

The structural characteristics of waste-derived carbon materials are not determined by feedstock composition alone. Processing route selection governs pore architecture, surface chemistry, crystallinity, and carbon yield to an extent that can override feedstock differences entirely [47,48]. A consolidated comparison of the principal processing routes and their structural outcomes is presented in Table 2, which provides a direct reference across pyrolysis, hydrothermal carbonisation, physical activation, chemical activation, and microwave-assisted processing. The subsections below discuss each route in terms of the structural outcomes it delivers and the parameters that govern them. Rice husk demonstrates how processing route governs structural outcome from the same precursor. Direct pyrolysis at 600–800 °C yields carbons with BET surface areas of 20–200 m^2^/g [88]. Hydrothermal carbonisation at 180–220 °C produces hydrochars with surface areas of 13–46 m^2^/g but higher oxygen functional group density, suited to surface-reactive adsorption [105]. KOH activation of rice husk char raises surface area to 1343–2364 m^2^/g depending on temperature and reagent ratio [106,107], with a predominantly microporous structure suited to CO_2_ capture and electrochemical storage. H_3_PO_4_ activation favours mesopore development, whilst ZnCl_2_ produces narrower micropore distributions at lower activation temperatures [57,62]. The same feedstock yields materials with markedly different structural and functional characteristics depending on processing route alone. The relationships between feedstock inputs, processing routes, intermediate products, and post-treatment pathways are shown schematically in Figure 2.

### 3.1. Pyrolysis

Pyrolysis is the thermal decomposition of organic matter under oxygen-limited or inert conditions and remains the most widely applied thermochemical route for producing carbon materials from waste feedstocks. The process yields three co-products, namely solid biochar, liquid bio-oil, and non-condensable gases, with relative proportions governed primarily by temperature and heating rate [47,48,49]. For carbon material production, the solid fraction is of principal interest, and its structural development is dictated to a greater degree by temperature than by any other single processing variable.

Temperature determines the phase of structural evolution in pyrolysis-derived carbons. At lower carbonisation temperatures (400–600 °C), biochar consists predominantly of amorphous carbon with abundant but undeveloped porosity [48,108]. As temperature increases toward and beyond 700 °C, progressive aromatisation and condensation of aromatic ring structures occur, transitioning the material through turbostratic char phases toward more ordered microcrystalline carbon [108]. The BET surface area of pyrolysis-derived carbons generally increases with temperature. Raw rice husk exhibits a surface area of only 2.2 m^2^/g, whilst pyrolysis at 600 °C yields a char with 141 m^2^/g. However, non-monotonic behaviour has been observed in some feedstocks where surface area decreases between certain temperature thresholds as pore coalescence or structural ordering reduces accessible micropore volume [109].

Heating rate determines the distribution between solid, liquid, and gaseous products rather than the intrinsic quality of the solid carbon. Slow pyrolysis, conducted at heating rates typically below 10 °C/min with extended residence times, maximises solid biochar yield by allowing more complete devolatilisation and structural reorganisation of the carbon matrix [47,48,49]. Biochars from slow pyrolysis exhibit substantially higher surface areas and more developed pore structures than fast pyrolysis counterparts. Fast pyrolysis switchgrass biochar typically achieves only 7.7 to 7.9 m^2^/g with almost no measurable porosity, whereas slow pyrolysis of the same material produces markedly higher values [48]. Fast pyrolysis, employing heating rates above 100 °C/s and short vapour residence times, is designed to maximise liquid bio-oil yield and is therefore less relevant to carbon material production as a primary objective, though the solid co-product can serve as an activation precursor.

Residence time governs the extent of pore network development. Extended residence times allow more complete volatilisation of hemicellulose and cellulose fractions, leaving behind a carbon matrix with progressively greater meso- and macroporosity [79]. Carrier et al. demonstrated directly that changes in pore structure during vacuum pyrolysis of sugarcane bagasse, specifically the development of channel networks and increased meso- and macropore distributions, have measurable effects on pyrolytic yields and downstream gasification reactivity [79].

Reactor configuration determines heat transfer uniformity and scalability. Fixed-bed reactors are widely used for slow pyrolysis due to their operational simplicity and precise temperature control, whilst fluidised-bed reactors provide the rapid and uniform heating required for fast pyrolysis at the expense of lower char yields [49]. Continuous-flow reactor configurations address the heat transfer and mass transfer limitations that emerge when moving from laboratory-scale batch systems to industrial operation. This transition changes temperature distribution and residence time uniformity in ways that bench-scale results cannot fully capture. This scalability dimension is examined further in Section 6.2.

### 3.2. Hydrothermal Carbonisation

Hydrothermal carbonisation occupies a distinct position among thermochemical conversion routes because it operates under conditions uniquely suited to wet feedstocks. It operates at moderate temperature with liquid water as the reaction medium and under autogenous pressure, eliminating the energy-intensive pre-drying step that conventional pyrolysis requires [5,50]. HTC is conducted at temperatures between 180 and 260 °C under subcritical water conditions, with pressures maintained above the saturation pressure of water at the reaction temperature. The fundamental advantage this confers is direct processability of high-moisture feedstocks such as sewage sludge, food waste, and wet agricultural residues without pre-treatment [5]. The reactions proceeding within the subcritical water environment include hydrolysis, dehydration, decarboxylation, condensation, polymerisation, and aromatisation, collectively transforming the organic feedstock into a carbon-enriched solid hydrochar alongside a chemical-rich process water [50].

Hydrochar differs structurally and chemically from pyrolysis-derived biochar in several important respects. Pyrolysis chars develop an ordered aromatic substructure through progressive decarboxylation and condensation at high temperature. Hydrochar, by contrast, retains considerably more oxygen functionality, exhibits higher local structural disorder, and is enriched with hydroxyl, carboxyl, and carbonyl surface groups [21,110]. This surface chemistry renders hydrochar more acidic and more hydrophilic than slow pyrolysis biochar, with implications for downstream activation and application [21].

As-produced hydrochar has a limited surface area, substantially lower than pyrolysis chars as reflected in Table 2, and requires activation to develop adequate pore structure for adsorption, catalytic, or electrochemical applications [51]. However, hydrochar responds differently to activation than pyrolysis-derived precursors. Pļavniece et al. demonstrated that NaOH activation of hydrothermally carbonised birch wood chips at 800 °C produced porous carbons with specific surface areas exceeding 2400 m^2^/g, comparable to pyrolysis precursors under similar activation conditions [51]. The critical distinction lies in pore architecture: HTC-derived activated carbons are consistently micro-mesoporous, whereas pyrolysis-derived activated carbons are predominantly microporous under equivalent activation conditions. This structural difference has practical consequences for mass transport and molecular access to active sites in electrocatalysis and adsorption applications [51,111].

Sequential HTC followed by pyrolysis and activation has emerged as a particularly effective strategy. Nieto et al. [112] demonstrated that HTC pretreatment prior to high-temperature pyrolysis increased the carbon content of the final material from 84–86% to 92–97% by XPS measurement. It simultaneously removed mineral impurities, specifically Mg, K, and Cl, that are retained in directly pyrolysed chars. Electrochemical performance of hard carbon anodes produced via this combined route exceeded that of directly pyrolysed materials by more than 100% at high charge–discharge rates [112,113].

### 3.3. Activation Methods

Activation transforms a carbonised precursor with limited surface area into a high-performance porous material by selectively removing carbon atoms and enlarging pore networks. It is not an optional refinement but in most cases a prerequisite for deploying waste-derived carbons in demanding adsorption, catalytic, or electrochemical applications [52]. Two principal strategies are employed. Physical activation uses oxidising gases; chemical activation uses reagent impregnation. They differ substantially in mechanism, operating conditions, the pore structures they generate, and their environmental burden, as summarised in Table 2.

Physical activation is a two-stage process in which the carbonised precursor is exposed to steam, carbon dioxide, or their mixture at temperatures typically between 800 and 1100 °C [52,54]. The gasification reactions selectively remove unstable carbon atoms that block pore entrances, progressively opening and enlarging the pore network. Steam and carbon dioxide operate through distinct reaction pathways. Steam proceeds via C + H_2_O → CO + H_2_, and CO_2_ via the Boudouard reaction C + CO_2_ → 2CO. Nevertheless, steam activation consistently achieves higher BET surface areas under comparable conditions. The smaller molecular size of water enables superior pore penetration, as demonstrated across bamboo (1182 vs. 911 m^2^/g), orange residue (388 vs. 248 m^2^/g), and paulownia (1166 vs. 800 m^2^/g) precursors [54]. Physical activation requires no corrosive reagents, has no washing steps, and generates no chemical-laden wastewater, and remains the dominant industrial production method where avoiding chemical procurement and wastewater management costs is a priority [53,114].

Chemical activation impregnates the precursor with a reactive agent prior to or during carbonisation, allowing simultaneous carbonisation and pore development at lower temperatures and shorter times than physical methods [52,54]. Potassium hydroxide is the most widely used activating agent and consistently produces the highest specific surface areas of up to 3700 m^2^/g under optimised conditions. These values are, however, obtained under optimised laboratory conditions using pre-selected precursors, and direct comparisons across studies are limited by differences in feedstock, KOH-to-carbon ratio, and activation temperature. KOH activation also carries practical drawbacks: it is a corrosive reagent, requires thorough acid washing of the product, and generates alkaline wastewater that must be treated before discharge [52,56]. The mechanism involves involving dehydration, redox reactions between KOH and the carbon framework (6KOH + 2C → 2K + 3H_2_ + 2K_2_CO_3_), and intercalation of metallic potassium into the carbon lattice [52,57]. Lower activation temperatures combined with high KOH-to-precursor ratios favour the formation of narrow micropores below 0.8 nm, which are particularly desirable for gas storage and CO_2_ capture. The aggressive nature of KOH activation introduces a significant post-treatment burden. Residual potassium compounds must be removed by acid washing and repeated rinsing, and the process generates corrosive wastewater. This practical and environmental cost that must be weighed against the superior surface areas achievable [52,54]. The volume of corrosive alkaline wastewater generated by KOH activation scales with reagent loading and requires neutralisation and treatment before discharge, adding operational cost and environmental burden not reflected in surface area comparisons [52,54]. H3PO4 activation presents lower toxicological and environmental concerns and is more suitable where environmental burden is a primary consideration, though at lower achievable surface areas [57].

Phosphoric acid activation operates at lower temperatures, typically around 500 °C, through a dehydrating mechanism in which phosphate ester cross-links form within the biomass structure, inhibiting structural shrinkage during carbonisation and promoting mesopore development [57]. H_3_PO_4_ presents lower toxicological and environmental concerns than ZnCl_2_ and is particularly effective for direct activation of uncarbonised lignocellulosic precursors [57,58]. Zinc chloride activation generates tunable micro-mesoporous structures. Pore character shifts from predominantly microporous at low impregnation concentrations to increasingly mesoporous at higher ratios, offering versatile pore architecture for applications such as supercapacitor electrodes, though at greater environmental cost than H_3_PO_4_ [57]. The choice between activation strategies should be guided by the target application, acceptable environmental burden, precursor composition, and economic context, with all of these dimensions captured comparatively in Table 2.

### 3.4. Microwave-Assisted Processing

Microwave-assisted carbonisation and activation have attracted considerable research interest as alternatives to conventional thermal processing, principally because of their fundamentally different heating mechanism and the structural consequences this produces. Conventional pyrolysis relies on conductive and convective heat transfer from the reactor wall inward. Microwave heating, by contrast, generates thermal energy volumetrically within the material itself through dielectric interactions between the electromagnetic field and polar molecular species [62,108]. This distinction produces carbon materials with measurably different structural characteristics from those achieved under conventional processing at nominally equivalent temperatures, as summarised in Table 3.

Most raw biomass materials are poor microwave absorbers, with dielectric loss tangent values below 0.1 [62]. Once carbonised above approximately 500 °C, however, the resulting char becomes an excellent microwave absorber through the Maxwell–Wagner effect and the displacement of π-electrons in carbonised structures [63]. Activating agents such as KOH and K_2_CO_3_ can improve the microwave absorbability of biomass precursors before significant carbonisation has occurred, enabling controlled initiation of the process [62,63].

The most consistently documented advantage of microwave processing is the dramatic reduction in processing time relative to conventional methods. Touhami et al. [61] demonstrated directly that microwave carbonisation of rice husk required 30 min compared to 4 h under conventional heating, and microwave sulphonation required 20 min versus 12 h. The microwave-prepared catalyst showed higher sulphur content (4.91% vs. 2.10%) and higher surface area (43.63 vs. 37.01 m^2^/g), demonstrating that time reduction is accompanied by quality improvement rather than compromise. More broadly, microwave heating has been shown to reduce processing times by up to 80% across a range of biomass treatments [116].

Carbon materials produced under microwave conditions consistently exhibit superior structural properties to those from conventional thermal processing. Chars from microwave pyrolysis demonstrate higher graphitic character as measured by Raman spectroscopy IG/Iall ratios, lower free radical concentrations, and higher BET surface areas. Biomass chars achieved 138 m^2^/g under microwave pyrolysis versus 71 m^2^/g under thermal pyrolysis in directly comparable experiments [115]. Microwave-assisted activation has achieved BET surface areas of up to 2996 m^2^/g. It produces ultra-microporous carbons with up to 73% of surface area from pores below 0.8 nm, a distribution particularly suited to CO_2_ capture, achieving uptakes of 5.3 mmol/g at 1 bar and 0 °C [62,63]. A distinctive feature of microwave pyrolysis not observed in conventional processing is the formation of carbon nanotubes and hollow carbon nanofibers on biochar particle surfaces. These are produced through a self-extrusion mechanism in which volatiles driven outward by the internal heating gradient resolidify on the particle surface [118].

These structural advantages carry practical limitations. Dielectric heterogeneity in mixed waste feedstocks produces non-uniform energy absorption and localised hot spots that create reproducibility challenges [96,98]. Microwave penetration depth constrains effective sample bed size, restricting batch capacity. No continuous industrial-scale microwave carbonisation system for waste-derived carbon production has been reported to date [62,119].

Microwave-assisted hydrothermal carbonisation of waste biomass produces hydrochars with properties that differ markedly from those obtained under conventional HTC at equivalent temperatures [120]. Process conditions including temperature, residence time, and biomass loading each exert distinct effects on hydrochar surface area, carbon content, and calorific value. A critical practical constraint is the challenge of achieving uniform temperature distribution. Hot spots arising from selective heating of polar regions in heterogeneous biomass materials can locally exceed target temperatures, creating reproducibility challenges that must be managed through careful reactor design [108,116]. Surface area development peaks at intermediate microwave power and declines at higher energies due to overheating and pore collapse [62,119]. The absence of industrial-scale demonstration units capable of continuous operation remains the principal constraint limiting microwave-assisted processing to laboratory and pilot scale, notwithstanding its structural advantages at those scales. This challenge is examined in Section 6.2.

## 4. Structural Characteristics of Waste-Derived Carbon Materials

**Figure 3:**
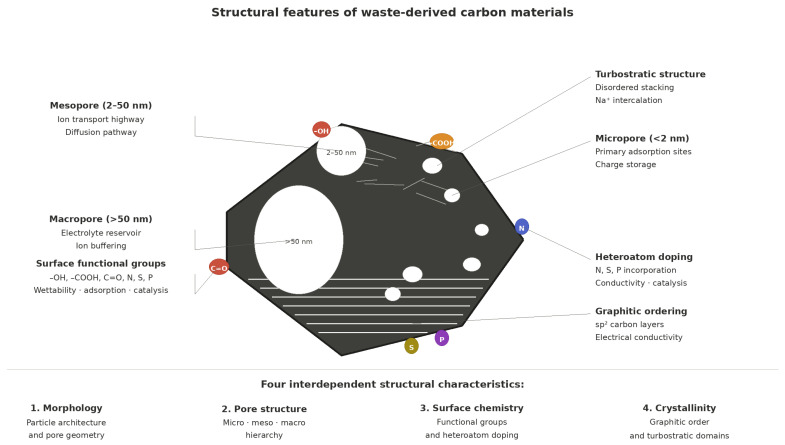
Schematic cross-section of a waste-derived carbon particle illustrating the four interdependent structural characteristics discussed in Section 4. The hierarchical pore architecture encompasses macropores (>50 nm) acting as electrolyte reservoirs, mesopores (2–50 nm) serving as ion transport highways, and micropores (<2 nm) providing primary adsorption and charge storage sites. Surface functional groups (–OH, –COOH, C=O) and heteroatom dopants (N, S) are surface-bound species that govern wettability, adsorption selectivity, and catalytic activity. Graphitic domains provide electrical conductivity whilst turbostratic regions accommodate Na^+^ intercalation. White ellipses represent pore cavities labelled by size. Coloured circles on the particle surface represent surface functional groups: red (–OH, C=O), orange (–COOH), blue (N), yellow-green (S), and purple (P). Grey lines indicate turbostratic carbon regions and white lines represent graphitic layers.

The functional performance of waste-derived carbon materials is governed by four interdependent structural characteristics: morphology and particle architecture, pore structure and surface area, surface chemistry and functional group composition, and crystallinity and graphitic ordering. These features are not independent outcomes of processing. They evolve together during thermochemical conversion in ways that reflect both feedstock chemistry and the specific processing route applied [47,48,121]. Understanding how they develop, how they are measured, and what their functional consequences are is central to the rational design of waste-derived carbons for targeted applications. The principal characterisation techniques used to probe these features, their key outputs, and their known limitations are consolidated in Table 4, which serves as a reference for the structural discussions that follow. The four structural characteristics and their principal functional roles are illustrated schematically in Figure 3.

### 4.1. Morphology and Particle Structure

The morphology of waste-derived carbon materials varies substantially with feedstock origin and is a primary determinant of bulk transport properties and adsorption behaviour [122,124]. Feedstock type determines porosity, apparent density, and carbon content of the resulting char. These properties propagate into the final product. Olive kernel biochar exhibited 0.862 g/cm^3^ apparent density and 43.6% total porosity, while pine sawdust biochar achieved 0.157 g/cm^3^ and 88.9% porosity under comparable conditions [144].

Wood-derived carbons retain structural features inherited from the vascular system of the plant precursor. SEM examination consistently reveals networks of channels and honeycomb-like architectures from preserved xylem and phloem structures. These create inherent fluid transport pathways, making wood-derived carbons well suited for water purification and gas adsorption [114,124]. Agricultural residue-derived carbons show heterogeneous morphologies that evolve with pyrolysis temperature. Sugarcane bagasse biochars transition from small surface holes at 300 °C to large irregular porosity at 600 °C, with BET surface areas of 180–198 m^2^/g, pore sizes of 1.42–4.33 nm, and pore volumes of 0.11–0.12 cm^3^/g [122,145]. Plastic-derived carbons present a distinctly different morphological picture. Synthetic polymer precursors lack any inherent cellular architecture, so pyrolysis generates spherical carbon structures through melt-flow and recrystallisation rather than preserving a biological matrix. Activation is therefore invariably required to introduce useful porosity [123,146]. Industrial waste-derived carbons, particularly those from sewage sludge, exhibit heterogeneous morphologies complicated by high inorganic content. Co-pyrolysis with carbon-rich biomass such as hazelnut shells markedly improves pore development by providing cellulose-rich carbon that drives surface area increase [95].

### 4.2. Porosity and Surface Area

Three terms used throughout this review require precise definition. Hierarchical porosity refers to carbon structures containing two or more distinct pore size regimes within a single material: micropores below 2 nm, mesopores of 2–50 nm, and macropores above 50 nm. This combination provides both high surface area and rapid ion transport pathways. Functional carbons are carbon materials whose performance in a target application is determined by specific structural or chemical characteristics rather than carbon content alone. Defect engineering refers to the deliberate introduction or control of structural irregularities in the carbon framework, including vacancies, edge sites, and heteroatom substitutions, to modify electronic structure, active site density, or ion intercalation behaviour.

Porosity is the structural feature most directly responsible for functional performance in adsorption, catalysis, and electrochemical applications. Pores are classified by the IUPAC convention into micropores below 2 nm, mesopores between 2 and 50 nm, and macropores exceeding 50 nm, each playing a distinct and non-interchangeable functional role [127,130]. Micropores are the primary contributors to specific surface area and serve as the main active sites for adsorption and electric double-layer charge storage. Mesopores function as ion transport highways, providing low-resistance diffusion pathways to micropore active sites and determining rate capability in electrochemical systems. Macropores act as electrolyte reservoirs that minimise ion diffusion distance under fast charge–discharge conditions [128,130]. Hierarchical pore structures integrating all three pore scales consistently outperform materials with uniform pore size distributions across adsorption, energy storage, and catalytic applications [127,130].

Optimal pore characteristics differ by application. For CO_2_ capture, pores below 1.0 nm are most effective, as confinement effects at this scale strengthen physisorption and carbons with pore widths below 1.0 nm outperform materials with larger pore widths [147]. For dye adsorption, mesopores and macropores are required to accommodate large organic molecules. Rhodamine B, with a molecular diameter of approximately 1.5 nm, shows substantially higher uptake in hierarchically porous carbons than in purely microporous equivalents under identical conditions [148]. For electrochemical double-layer capacitors, optimal micropore widths of approximately 0.7 nm have been reported for aqueous electrolytes and 0.8 nm for organic electrolytes, reflecting the partial desolvation of ions required for pore entry [147,149]. For sodium-ion battery anodes, micropores in the 0.4–2 nm range support pseudocapacitive sodium storage at pore surfaces and defect sites [150]. Mesopores of 2–6 nm serve the complementary role of facilitating ion transport to these active sites [151].

Chemical activation of waste-derived precursors has generated some of the highest surface areas reported for carbon materials. Waste bamboo-derived hierarchical porous carbons achieved 3392 m^2^/g with pore volumes of 2.081 cm^3^/g through combined H_3_PO_4_ hydrothermal pretreatment and KOH/melamine co-activation [152]. Seaweed-derived hierarchical porous carbonaceous aerogels achieved 2200 m^2^/g [128]. Coconut shell biochar, a benchmark precursor for its high lignin content and dense framework, produced 1252 m^2^/g with a type I BET isotherm confirming a predominantly microporous structure [114]. These surface area values carry a structural trade-off. Extensive KOH etching progressively thins pore walls, reducing mechanical strength and increasing susceptibility to pore collapse during compression or repeated electrochemical cycling. Very high surface areas do not therefore guarantee superior functional performance, as pore wall integrity and connectivity become limiting factors.

A critical caveat accompanies the interpretation of BET surface area in highly microporous carbons. The BET model was developed for multilayer adsorption on relatively flat surfaces and does not accurately describe the volume-filling mechanism that governs adsorption in micropores, meaning that measured surface area does not reliably reflect functional accessibility [125,126]. Activated carbons with BET surface areas approaching 3000 m^2^/g have been reported to perform worse than materials with lower but more accessible surface areas. High pore tortuosity and poor connectivity severely limit electrolyte ion transport regardless of nominal surface area [126]. Pore connectivity is one of the most critical yet underappreciated structural parameters. Hierarchical porous carbon nanospheres with the lowest pore separation distance of 10 nm delivered 405 F/g at 1 A/g with 71% retention at 200 A/g, showing that connectivity directly translates into high-rate performance [132]. Complementary techniques are therefore necessary adjuncts to BET nitrogen adsorption for complete characterisation of microporous carbons. These include CO_2_ adsorption at 273 K for narrow micropores, n-nonane pre-adsorption for separating micro- and mesopore contributions, and argon adsorption at 87.3 K [125,126].

### 4.3. Surface Chemistry and Functional Groups

The surface chemistry of waste-derived carbon materials governs wettability, adsorption selectivity, catalytic activity, and pseudocapacitive charge storage in ways that pore structure alone cannot determine [133,135,153]. Oxygen-containing functional groups, principally hydroxyl, carboxyl, and carbonyl species, are particularly abundant on biomass-derived and hydrothermally produced carbons, where the oxygen content of the lignocellulosic precursor is partially retained. These groups increase surface affinity for aqueous electrolytes and polar contaminants, contribute pseudocapacitive charge storage through reversible faradaic reactions, and provide active sites for heavy metal complexation and ion exchange [135,154]. Pyrolysis temperature exerts a decisive influence. Lower temperatures preserve a greater proportion of oxygen functionalities, while higher temperatures promote deoxygenation and graphitisation. This trade-off between surface reactivity and electronic conductivity must be optimised for each target application [138,154].

Heteroatom doping, the incorporation of nitrogen, sulphur, phosphorus, or boron into the carbon framework, provides a more targeted route to modifying electronic structure, conductivity, and catalytic activity [135,139,154]. Nitrogen doping adopts multiple bonding configurations including pyridinic-N, pyrrolic-N, and graphitic-N, each contributing distinctly to electrochemical performance. Pyridinic-N and graphitic-N are generally the most active for electrocatalytic oxygen reduction, while all configurations enhance conductivity and introduce basic sites beneficial for energy storage [136,137]. These differences reflect distinct electronic roles. Pyridinic-N donates one electron to the π system, creating Lewis base sites that activate molecular oxygen in ORR [137]. Graphitic-N substitutes into the basal plane and raises the Fermi level, improving conductivity without creating discrete active sites [136,137]. Pyrrolic-N, incorporated into five-membered rings, contributes to pseudocapacitive charge storage through reversible redox reactions [136]. Sulphur and phosphorus doping modify electronic charge distribution and interlayer spacing in complementary ways, and their co-incorporation with nitrogen produces synergistic effects that exceed single-element doping in both capacitive and electrocatalytic performance [138,140,155]. Biomass-derived carbons are particularly amenable to self-doping strategies. Nitrogen-rich precursors such as orange peel have been converted into nitrogen and sulphur co-doped materials through direct pyrolysis routes, demonstrating that precursor chemistry can be harnessed directly without separate chemical treatment [31,156]. Surface chemistry is characterised primarily by FTIR spectroscopy for functional group identification and XPS for quantitative elemental composition and bonding configuration analysis [136,154].

### 4.4. Crystallinity and Graphitic Structure

The degree of crystallinity and graphitic ordering in waste-derived carbon materials directly determines electrical conductivity, thermal stability, and suitability for electrochemical energy storage [143,157]. Carbon materials span a structural spectrum from fully amorphous phases through turbostratic carbon to highly crystalline graphite. Turbostratic carbon has graphene-like layers stacked in parallel but with random rotational disorder, while graphite has three-dimensional AB stacking order and an interlayer d_002_ spacing of 3.35 Å [141,158]. Turbostratic carbon exhibits interlayer spacings of 3.42–3.50 Å, reflecting the rotational disorder that simultaneously expands interlayer distance and suppresses electronic coupling between adjacent layers [141,159].

XRD and Raman spectroscopy are the two most widely employed and complementary techniques for characterising crystallinity, each probing different and non-equivalent structural features [157,160]. In Raman spectroscopy, the G band at approximately 1580 cm^−1^ reflects sp^2^ C-C stretching in all graphitic carbon. The D band at approximately 1350 cm^−1^ is a defect-activated mode. Its intensity relative to the G band, the ID/IG ratio, serves as the primary quantitative indicator of structural disorder [157,161,162]. The 2D band at approximately 2700 cm^−1^ provides definitive information on stacking order, appearing as a single symmetric peak in turbostratic carbon and splitting into asymmetric subpeaks upon development of AB stacking [114,141,143]. In XRD, the position and breadth of the (002) reflection directly reflect interlayer spacing and structural ordering along the c-axis. The Scherrer equation estimates crystallite dimensions, though Lc and La values from XRD measure different structural characteristics than those from Raman spectroscopy and should not be directly compared [142,157].

Carbonisation temperature is the primary driver of structural evolution. Progressive heating removes heteroatoms and grows aromatic domains. In graphitisable soft carbons, it eventually generates three-dimensional stacking order. Hard carbons retain turbostratic structure even above 3000 °C due to structural cross-linking between graphene layers [143,157,163]. This distinction between hard and soft carbons is fundamental for sodium-ion battery applications. Hard carbons from biomass waste provide the expanded interlayer spacing needed to accommodate Na^+^ ions alongside microporous and mesoporous architecture. This enables combined intercalation, adsorption, and pore-filling storage, delivering discharge capacities of up to 450 mAh/g with stable cycling over 250 cycles [14,142]. Catalytic graphitisation using base metal nanoparticles enables graphitic structure formation at temperatures as low as 715–800 °C, well below the threshold for thermal graphitisation. The onset is confirmed by the appearance of sharp (002) XRD reflections and characteristic changes in the Raman D/G band profile [163,164]. Higher graphitic ordering directly enhances electrical conductivity. Highly ordered graphene films achieved approximately 2.0 × 10^4^ S/cm, a sixfold improvement over disordered counterparts. However, this comes at the cost of reduced surface functional groups and potentially reduced ion accessibility, reinforcing that the optimal degree of graphitisation must be application-specific [114,141].

The structural features discussed across Section 4.1, Section 4.2, Section 4.3 and Section 4.4 and their functional consequences are consolidated in Table 4, which provides a concise cross-reference between structural parameters, their characteristic ranges, and their performance implications across the principal application domains addressed in Section 5.

## 5. Structure–Property Relationships in Waste-Derived Carbon Materials

The structural characteristics discussed in Section 4, namely morphology, pore architecture, surface chemistry, and crystallinity, do not govern performance independently. Their functional consequences emerge from the relationships between them, and understanding these relationships is what separates rational material design from empirical trial and error [48]. Porosity and surface area are necessary but not sufficient for high performance. Surface functional groups must be matched to the target application. Pore size distribution must accommodate the molecular dimensions of target species, and graphitisation degree must be balanced against active site density and functional group retention [48,165,166]. The following subsections examine how these structural parameters quantitatively govern performance across the four principal application domains of waste-derived carbon materials: electrochemical energy storage, adsorption, catalysis, and high-temperature structural deployment.

### 5.1. Electrical and Electrochemical Properties

The electrical conductivity of waste-derived carbon materials spans an extraordinarily wide range, from 10^−8^ to 10^10^ S/m depending on degree of graphitic ordering. This range directly determines suitability for electrochemical applications [166]. Graphitisation degree is the primary structural determinant of conductivity: higher carbonisation temperatures promote the formation of extended sp^2^ carbon networks with reduced interlayer spacing, enhancing electronic transport through the material [135,167]. However, this relationship is not monotonic with respect to electrochemical performance, because the same graphitisation process that improves conductivity simultaneously reduces surface area, eliminates surface functional groups, and lowers defect density. These are the very structural features that provide active sites for ion adsorption and charge storage [135,168,169].

The resolution of this tension lies in the concept of optimal defect density. Jia et al. [168] demonstrated quantitative correlations between defect density, characterised by the mean distance between defects from Raman spectroscopy, and electrochemical activity, establishing that a moderate defect density range provides the best combination of active site availability and electronic conductivity. Zhong et al. [170] corroborated this by showing that the optimal heterogeneous electron transfer rate in single-layer graphene occurs at a moderate defect density where the material is electronically activated yet maintains structural integrity. Yuan et al. [171] quantified the relationship directly for supercapacitor applications. Self-doping defects provide approximately 90 F/g per unit of defect density as measured by the AD/AG ratio from Raman spectra, with optimised graphene blocks achieving 52.2% capacitance retention at 20 A/g. Hierarchical porous carbons integrating optimised defect density with micro-meso-macropore architecture have achieved specific capacitances as high as 612 F/g at 5 mV/s [135].

For sodium-ion battery anodes, an application of direct relevance to waste-derived hard carbons, the structural requirements differ markedly from those of supercapacitors. Hard carbons, which retain turbostratic structure with interlayer spacings typically above 3.7 Å even at high processing temperatures, provide the expanded interlayer distance required for Na^+^ intercalation that is thermodynamically unfavourable in well-ordered graphite [167,172]. Turbostratic domains also contain vacancy defects and edge sites that serve as supplementary Na^+^ adsorption sites beyond intercalation, with divacancy defects, as shown by DFT calculations, more effective than monovacancy defects in improving Na adsorption energy [169]. Pre-carbonisation at moderate temperatures stabilises the precursor structure and refines micropore volume, whilst post-treatment hydrogen reduction reduces structural defects and oxygen functional groups, together enabling greater than 90% capacity retention over 200 charge–discharge cycles in optimised hard carbon anodes [173].

Co-engineering of heteroatom doping with controlled defect introduction represents the most powerful design strategy for simultaneously optimising conductivity, active site density, and ion storage capacity. Both dopants and structural defects modulate charge and spin distribution at the electronic structure level, and their synergistic effects on ion transport and active site density exceed the sum of their individual contributions. This principle has been demonstrated across supercapacitors, lithium-ion batteries, and potassium-ion systems [174,175,176].

### 5.2. Adsorption Performance

Adsorption performance in carbon materials is governed by three structural parameters acting in concert. Pore size distribution determines both capacity and kinetic accessibility. Surface functional groups govern selectivity and interaction strength. Pore connectivity determines whether theoretical surface area translates into practical adsorption under operational time constraints [48,177,178]. The dominant interaction mechanism varies with adsorbate chemistry. Cationic dyes interact through electrostatic attraction with negatively charged surface groups and through π-π stacking between their aromatic rings and the graphitic carbon basal plane [179,180]. Heavy metal cations are retained through ion exchange and complexation with nitrogen and oxygen functional groups [179,181]. Polar organic contaminants engage through hydrogen bonding with hydroxyl and carboxyl surface species, whilst non-polar compounds are removed primarily through hydrophobic interactions within the pore network [179]. Solution pH governs adsorption performance through its effect on both adsorbate speciation and carbon surface charge. Below the point of zero charge (pHpzc), the carbon surface carries a net positive charge favouring anionic species, whilst above pHpzc the surface becomes negatively charged and preferentially attracts cationic contaminants such as methylene blue and heavy metal ions. Systematic reporting of pHpzc values alongside performance data would improve comparability across studies.

The pore size–adsorbate size relationship is the most fundamental determinant of adsorption capacity. For small gas molecules, micropores, and specifically ultramicropores below 0.7 nm, are the decisive structural feature. Pores smaller than 2.02 nm were identified as the dominant contributors to CO_2_ uptake in phosphorus-doped porous carbons, with LPSP-700 achieving 2.51 mmol/g at 25 °C and 1 bar, with nearly 90% of equilibrium uptake reached within 6 min [182]. Nitrogen-autodoped hierarchical porous carbons achieved CO_2_ adsorption capacities of 5.0 mmol/g, a 230% increase over the precursor material, through the combination of hierarchical micro-meso-macroporosity and nitrogen defects providing chemisorption sites complementary to physical adsorption in micropores [183]. The coexistence of micropores for capacity and mesopores for transport kinetics improves molecular diffusivity by at least one order of magnitude compared with purely microporous materials [184].

For the adsorption of large organic molecules from aqueous solution, broader pore size distributions become essential. Rhodamine B, with a maximum molecular diameter of approximately 1.5 nm, achieves optimal removal when mesoporous and macroporous channels are available to reduce entrance resistance. Hierarchically porous activated carbon achieved 881 mg/g adsorption capacity, compared with only 374 mg/g for microporous activated carbon under identical conditions [148]. The most striking reported performance for dye removal illustrates the potential of this approach. A rhodamine B adsorption capacity of 9444 mg/g was achieved from O–N–S self-doped hierarchical porous carbon derived from lotus leaves, with a surface area of 3601 m^2^/g. This illustrates how hierarchical pore architecture and heteroatom-enhanced wettability act together to amplify both the accessible surface area and the adsorption driving force [185].

Heavy metal adsorption introduces the additional dimension of surface chemistry selectivity. Nitrogen-autodoped porous carbons achieved a Langmuir adsorption capacity of 434.8 mg/g for Hg(II), with DFT calculations confirming that performance arises from chemisorption at nitrogen defect sites operating in concert with physical adsorption through the hierarchical pore network. Neither structural feature alone accounts for the full result [183]. Nitrogen-doped hierarchical porous biochar from corn stalks demonstrated that phenol adsorption capacity is positively correlated with graphitic-N content specifically, because graphitic nitrogen forms π–π bonds with phenol molecules that enhance adsorption beyond what pore filling alone achieves [181].

Adsorption kinetics follow pseudo-second-order models across virtually all reported systems, consistently indicating that chemisorption or chemical interaction, rather than simple physical filling, is the rate-limiting step [178,183,185]. Multi-stage intraparticle diffusion is characteristic of hierarchical porous carbons, with rapid initial diffusion through macropores and mesopores followed by slower penetration into micropores. This two-stage kinetic profile confirms that the structural design has successfully separated transport function from storage function. Macropores and mesopores handle rapid bulk transfer while micropores provide the high-density adsorption surface. This combined function is absent in purely micro- or purely mesoporous carbons [148,179,186].

External film diffusion represents an additional rate-limiting step under conditions of low agitation or high carbon loading, yet it is rarely characterised in laboratory batch studies [179,186]. In continuous flow systems, kinetic performance rather than equilibrium capacity determines removal efficiency, a distinction that batch data alone cannot capture.

The adsorption data reviewed here derive predominantly from batch systems. Fixed-bed column studies introduce additional performance determinants including breakthrough behaviour, pressure drop, regeneration efficiency, and long-term stability under continuous operation. Such data are not well represented in the waste-derived carbon literature reviewed here, and this gap warrants attention in future work.

Adsorption thermodynamics provide a further dimension not systematically covered in this review. Van’t Hoff analysis of temperature-dependent equilibrium data consistently yields negative ΔG° values confirming spontaneous adsorption [187,188]. Enthalpy values vary by adsorbate; dye adsorption is commonly endothermic (ΔH° = +23.54 kJ/mol for methylene blue on biochar [188]), whilst heavy metal adsorption can be exothermic. Positive ΔS° values are widely reported, reflecting increased disorder at the solid-solution interface [187,188].

The adsorption performance data reviewed here derive predominantly from single-component systems. In real wastewater streams, competitive adsorption between co-existing contaminants reduces uptake of individual species relative to single-component values, with the extent of competition depending on relative concentrations, molecular size, and affinity for available surface sites. This distinction is not yet systematically addressed in the waste-derived carbon literature and represents a further gap between laboratory characterisation and practical deployment.

### 5.3. Catalytic Activity

The catalytic performance of waste-derived carbon materials is governed by a structural hierarchy in which surface area and pore volume are enabling conditions, but active site accessibility and surface chemistry are the decisive determinants of activity [189,190]. This principle is best illustrated by xylan hydrolysis. Biochar achieved higher conversion (85 vs. 57%), an initial reaction rate 30 times higher, and a turnover frequency 9 times higher than activated carbon, despite a substantially lower surface area (365 vs. 1391 m^2^/g). The energy-intensive activation process that generates the high surface area of activated carbon simultaneously destroys the sulfonation sites necessary for catalytic activity [189]. Active site density and accessibility, rather than total surface area, is therefore the key structural parameter for carbon catalysts.

Pore architecture mediates this accessibility in application-specific ways. For reactions involving large organic molecules such as long-chain fatty acids in biodiesel production, macroporous structures outperform mesoporous ones. Sulfonated activated carbon with more macropores achieved 74.47% FAME conversion compared with 46.98% for a predominantly mesoporous catalyst, because large pore sizes support the diffusion of bulky substrate molecules to active sites [191]. The rate-limiting step shifts with pore architecture in a way that must be matched to the molecular dimensions of the reaction system [190].

For biodiesel production from waste feedstocks with high free fatty acid content, sulfonated carbon catalysts provide a quantitatively instructive example of structure–activity relationships. Catalytic activity depends on the synergistic action of –SO_3_H groups as Brønsted acid sites, supported by –OH and –COOH groups that function as additional adsorption sites for substrate molecules. The combined effect of these groups, rather than –SO_3_H density alone, accounts for the high total acid density and catalytic performance [192,193]. Biomass-derived sulfonated carbon catalysts consistently exceed 90% biodiesel yield. Peanut shell-derived catalysts achieved 94.91% yield with 79.85% retention after five cycles [193]. Camellia oleifera shell biochar achieved 91.4% yield with over 90% retention after four cycles [194]. Superhydrophobic spherical activated carbon catalysts maintained 86.8% yield in the tenth cycle by preventing water-induced deactivation of acid sites [195]. The fundamental design challenge is the trade-off between carbonisation temperature, surface area, and acid site density: higher temperatures produce more graphitic carbon with greater surface area but progressively reduce total acid density [196].

Beyond solid acid catalysis, structural defects serve as primary active sites for a broader range of reactions. Metal-loaded carbon catalysts exploit biochar surface functional groups as anchoring sites for metal precursors. Impregnating 3 wt% MnO_x_ onto rice straw biochar achieved 84% NO_x_ removal at 50 °C [189]. Nitrogen-doped carbons with pyridinic-N and graphitic-N configurations provide active sites for electrochemical oxygen reduction. Activity approaches that of commercial Pt/C in certain electrolyte systems [197]. Carbon scaffolds also bridge radical and non-radical oxidation pathways in advanced oxidation processes, with defect sites and surface functional groups governing reaction selectivity [105]. Porous carbon materials show activity in photothermal CO_2_ reduction, with broad-spectrum light absorption and π–π interactions enabling dispersion of active phases [107].

### 5.4. Thermal Stability and Mechanical Integrity

The thermal stability and mechanical integrity of waste-derived carbon materials are structural properties whose importance is often underweighted in laboratory studies focused on surface area and adsorption capacity. They become decisive in applications involving elevated temperatures, repeated thermal cycling, or sustained mechanical loading [198]. Both properties are governed by graphitic ordering, and both are subject to the same fundamental trade-off: increasing graphitisation improves stability but reduces the surface area, porosity, and functional group density that enable functional performance [198,199].

Oxidation resistance scales directly with graphitisation degree. Graphitic carbon coatings on metal oxide nanoparticles showed oxidation onset temperatures varying from 361 °C to 463 °C depending on graphitisation level, with more ordered structures exhibiting higher onset temperatures [200]. Raman graphitisation indices, specifically D/G, G’/G, and G’/D intensity ratios, correlate directly with the apparent activation energy for oxidative degradation in carbon nanotubes, confirming that crystalline perfection translates into measurable thermal resistance [201]. In electrochemical environments, graphitisation treatment of ordered mesoporous carbons at 1500–2000 °C reduced corrosion rates at 1.2 V vs. RHE compared with commercial Vulcan carbon black, whilst preserving mesopore architecture. This demonstrates that carefully selected graphitisation conditions can balance structural order with textural properties [198]. Catalytic graphitisation of waste biomass using manganese nitrate below 1000 °C enables graphitic structure formation without the extreme temperatures typically required, with graphitisation degree dependent on catalyst concentration and treatment temperature [202].

The H/C and O/C elemental ratios serve as practical indicators of aromaticity and thermal stability in waste-derived carbons: lower values in both cases indicate more condensed aromatic structures associated with greater thermal resistance [165,202]. Excess surface oxygen functional groups, whilst beneficial for wettability and adsorption, weaken the carbon framework at elevated temperatures. This is a practical constraint that acid treatment conditions must respect if thermally stable products are to be achieved [176,198]. For hard carbon anodes in sodium-ion batteries, pre-carbonisation strategies that refine micropore volume without substantially altering graphitisation level improve both cycling stability and structural resilience, contributing to the greater than 90% capacity retention over 200 cycles reported for optimised systems [173].

### 5.5. Synthesis: Cross-Cutting Structure–Property Principles

**Figure 4:**
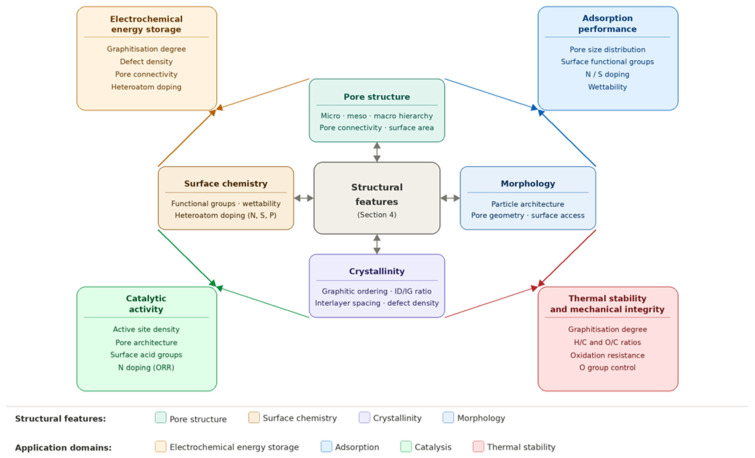
Structure–property relationships in waste-derived carbon materials. The central hub represents the four structural features discussed in Section 4. Double-headed arrows indicate the bidirectional relationship between hub and structural features; single-headed coloured arrows show which structural features govern each application domain. Key structural determinants for each domain are listed within the application boxes. ORR = oxygen reduction reaction.

The evidence across Section 5.1, Section 5.2, Section 5.3 and Section 5.4 converges on a set of principles that apply regardless of application domain. Porosity and surface area are necessary but not sufficient: surface functional groups must be matched to the target species and interaction mechanism, and pore size distribution must accommodate the molecular dimensions of the target [48,166]. Graphitisation degree and surface functionality are competing requirements that must be balanced for the specific application. Higher conductivity is needed for electrochemical systems, whilst higher functional group density is needed for adsorption and catalysis [48,203]. Pore connectivity determines whether theoretical surface area translates into practical performance under operational conditions [131,132]. Heteroatom doping and structural defects are most powerful when co-engineered rather than introduced independently, because their synergistic effects on charge distribution, active site density, and ion transport exceed the sum of their individual contributions [174,175]. Finally, the feedstock and processing route selections made in Section 2 and Section 3 determine the structural space that is accessible: no post-production modification can compensate for a mismatched precursor-process combination [165]. These principles provide the rational design framework that connects the processing routes of Section 3, the structural features of Section 4, and the performance outcomes summarised quantitatively in Table 5. This framework is summarised visually in Figure 4.

The evidence reviewed supports the following feedstock-processing combinations for specific applications. For CO_2_ capture, high-lignin biomass with KOH activation produces the narrow micropore distributions required [52,147]. For dye adsorption, agricultural residues with H3PO4 activation yield mesopore-rich structures that accommodate large molecules [89,168]. For supercapacitor electrodes, nitrogen-rich precursors processed by pyrolysis provide the conductivity and pseudocapacitance required [127,128]. For sodium-ion battery anodes, cellulose-rich biomass pyrolysed at high temperature without activation produces the defect-rich turbostratic structure needed for Na+ intercalation [150,175].

Performance values cited across Section 5.1, Section 5.2, Section 5.3 and Section 5.4 and in Table 5 should be interpreted with caution, as adsorption capacity, CO_2_ uptake, and capacitance figures originate from studies using different carbon doses, adsorbate concentrations, temperatures, and electrolyte compositions. Direct numerical comparison across systems is not valid without normalisation of these experimental conditions.

Material regeneration and cycling stability are not systematically covered across all application domains in this review. Catalytic performance data, including retention of approximately 80% over five cycles [193] and above 90% over four cycles [194] for sulfonated carbon catalysts, are discussed in Section 5.3. Equivalent multi-cycle characterisation for adsorption applications remains limited in the waste-derived carbon literature and warrants systematic attention in future work.

## 6. Challenges, Scale-Up Considerations, and Future Perspectives

The translation of waste-derived carbon materials from laboratory demonstration to reliable industrial production requires confronting a set of interconnected challenges that span feedstock management, process engineering, environmental compliance, economic viability, and the foundational standards infrastructure that underpins all of these. Each challenge is tractable, but none is trivial. Their resolution will determine whether the performance documented in Section 4 and Section 5 is ever realised at commercially meaningful scale.

### 6.1. Feedstock Variability and Material Reproducibility

Feedstock variability is the most pervasive challenge in waste-derived carbon material production because it propagates into every downstream step of processing, characterisation, and application [5,204]. Lignocellulosic biomass, the most widely used precursor category, varies in its relative proportions of cellulose, hemicellulose, and lignin depending on plant species, growing conditions, harvest time, and geographic origin. Rice husk alone spans 70–80% organic content alongside 20–30% mineralogical components including silica and alkali metals [7,13]. Even within a single feedstock type, seasonal fluctuations in composition translate directly into inconsistent carbon yields and structural properties when process parameters are held constant [205,206]. Not all waste biomass feedstocks are equally amenable to carbonisation. Of multiple agricultural wastes evaluated by Skoczko and Gumiński [207], only sugar beet fibre marc produced valuable activated carbon, while cherry stones and mixed vegetable waste yielded materials with unacceptably high moisture, high ash, and low surface area.

Plastic waste streams present a qualitatively different variability challenge. Real-world mixed plastic waste comprises LDPE (23%), HDPE (19%), PP (14%), PS (9%), PET (10%), and PVC (6%) among others. Each polymer type has markedly different thermal decomposition behaviour and carbon content ranging from 65 to 98% [208,209]. PVC requires particular management: its dominant chlorine content introduces risks of reactor corrosion, chlorinated compound formation at high temperature, and secondary contamination of carbon sorbent products, requiring its exclusion from mixed-stream processing or dedicated pre-treatment [209,210]. Sewage sludge composition varies with catchment area, treatment process, industrial inputs, and seasonal factors, with the variable heavy metal content representing a critical quality control parameter that directly determines the suitability of derived carbons for environmental or agricultural applications [5].

Practical mitigation strategies include feedstock blending to achieve more consistent compositions, process parameter adaptive control to compensate for batch-to-batch variation, and source control measures that address variability at the point of generation rather than post-collection [208]. These include the valorisation of unconventional polymer waste streams, such as surgical masks, as activated carbon precursors. Quality standards for waste-derived carbon materials remain underdeveloped. The European Biochar Certificate and related frameworks provide partial guidance for agricultural applications but do not address the full range of structural and chemical properties relevant to energy storage, catalysis, or high-specification adsorption applications [211]. Developing internationally recognised quality standards is a prerequisite for market integration and one of the most pressing near-term needs in the field.

### 6.2. Process Scalability and Energy Requirements

The transition from batch laboratory synthesis to continuous industrial production requires reactor configurations capable of handling heterogeneous feedstocks reliably while maintaining the structural consistency that functional applications demand. Rotary kiln reactors are the most mature and widely deployed technology for continuous pyrolysis at an industrial scale. Their inclined rotating cylindrical design enables continuous feedstock transport, flexible control of residence time through rotation speed and kiln slope, and the ability to process feedstocks with irregular particle sizes without extensive pre-treatment [35,212]. Commercial waste tire pyrolysis operations have demonstrated rotary kiln production at approximately 24 tonnes per day, confirming that continuous operation with appropriate process economics is commercially viable [213]. Pyrolytic char from continuous rotary kiln processing achieves BET surface areas of approximately 89 m^2^/g directly after carbonisation, rising to 306 m^2^/g after CO_2_ activation at 51.3% burnoff, confirming the route to high-performance activated carbon from a continuous industrial process [214]. The primary shortcoming of rotary kilns for carbon material production is lower heat transfer efficiency compared with fluidised bed systems, which can result in incomplete thermal processing at high throughput [35,215]. At larger scale, radial temperature gradients in fixed-bed and rotary kiln reactors mean that carbon particles at different positions experience different peak temperatures and heating rates. This produces variation in pore structure and surface chemistry within a single production batch that small homogeneous laboratory samples do not reflect.

Fluidised bed reactors offer superior heat and mass transfer and are the most readily scalable for fast pyrolysis applications targeting bio-oil co-production, with industrial demonstrations reaching 200 tonnes per day [216,217]. Auger and screw reactors provide a complementary option for continuous carbon material production with minimal carrier gas requirements and modular scalability, exemplified by the Carbo twin retort system deployed commercially across multiple continents [217,218]. Microwave pyrolysis offers energy recovery improvements, rising from 84% for conventional processing to 99% in some demonstrations. Industrialisation remains constrained by engineering challenges in managing microwave leakage during continuous operation and ensuring uniform heating with variable feedstock compositions [219,220].

The energy economics of scale-up are significant. Producing biochar requires 1.1–16 MJ/kg, while activated carbon demands approximately 44–170 MJ/kg, roughly ten times higher, reflecting the energy-intensive activation step that distinguishes functional carbon material production from simple charring [221]. Autothermal operation, where process energy derives entirely from pyrolysis co-products including tar and non-condensable gases, has been demonstrated at pilot scale in a continuous plant processing 2000 kg/h. This represents a technically important proof of concept for energy self-sufficient industrial production [222].

### 6.3. Environmental and Regulatory Considerations

The environmental credentials of waste-derived carbon materials are well established. Benefits include waste diversion from landfill, reduced dependence on fossil-derived activated carbon, carbon sequestration potential, and substitution of conventional carbon black and graphite with sustainable alternatives. These benefits are only fully realised when process emissions and product contaminants are adequately managed [223,224]. Pyrolysis of solid waste and biomass generates a complex mixture of air pollutants including volatile organic compounds, polycyclic aromatic hydrocarbons, particulate matter, NO_x_, SO_x_, and CO, with systematic mapping identifying 25 distinct air pollutants from pyrolysis plant operations [224,225]. VOC formation from biomass pyrolysis follows the sequential degradation of functional groups: acids, then phenols and esters, then alcohols and aldehydes, then hydrocarbons and aromatics. Formation is strongly influenced by feedstock composition, temperature, and heating rate [226,227]. Biochars produced at temperatures above 400 °C with H/C ratios below 0.70 do not release VOCs at ambient temperatures, providing a practical process criterion for producing safe products. Low-temperature biochars, by contrast, can contain over 140 sorbed VOC species and may exceed occupational exposure limits during handling and storage [226,228,229].

Effective emission control technologies exist and have been demonstrated at scale. Scrubbing systems combining hot char filtration, condensation, oil scrubbing, and alkaline solution treatment have been shown to remove up to 98% of tar and substantial fractions of particulate matter and heavy metal vapours from MSW pyrolysis gas [230]. Afterburners reduce volatile pyrolysis products by at least 80%, though with the trade-off of increased CO_2_ emissions [231]. The EU Industrial Emissions Directive and US Clean Air Act provide regulatory frameworks, though jurisdictional inconsistencies in classifying pyrolysis operations relative to incineration create permit uncertainty that impedes commercial deployment [224,232]. Persistent data gaps represent priority areas for regulatory-grade research, particularly regarding volatile fluorochemical emissions from pyrolysis of PFAS-impacted biosolids and the long-term heavy metal leaching behaviour of sewage sludge-derived chars [233,234]. Life cycle assessment integrating all emission streams and contaminant pathways alongside the positive contributions of waste valorisation is essential for establishing the genuine environmental balance of waste-derived carbon material production systems [223,235].

### 6.4. Economic Feasibility and Market Integration

The cost competitiveness of waste-derived biochar relative to commercial activated carbon is well established. Biochar electrode materials cost 54–401 € t^−1^ compared with 843–2635 € t^−1^ for granular activated carbon and 527–843 € t^−1^ for graphite granules. Wood-derived biochar at $91–329 per tonne compares favourably against activated carbon at approximately $1500 per tonne [236,237]. Techno-economic analysis of activated carbon production from pea waste estimated an internal rate of return of 55% and return on investment of 52% with cost recovery from year three. The authors noted the absence of established industrial-scale biomass-to-activated-carbon plants as a key limitation of current knowledge [238]. The energy cost differential between biochar and activated carbon production is approximately tenfold. The economic case for higher-specification carbon materials therefore rests heavily on achieving premium product valuations through demonstrated performance in target applications [221].

Standalone biochar production remains largely unprofitable under most market conditions without supplementary revenue streams. In one German analysis, the only break-even scenario required a carbon removal price of at least 72 €/t CO_2_eq on voluntary carbon markets [239]. The most economically viable configurations integrate biochar production into biorefinery frameworks co-producing bio-oil, syngas, and biochemicals alongside the carbon material. One NPV-based comparison showed co-production revenue of $24.2 million versus $5.5 million for a waste management scenario alone [240]. Carbon credit mechanisms, governmental incentives, and embedding biochar carbon removal within official regulatory frameworks are consistently identified as essential enabling conditions for commercial viability at scale [239,241,242]. The absence of industrial-scale production facilities and dependence on extrapolated techno-economic assumptions remains the principal gap between current knowledge and investment-grade commercial decision-making [238,243]. Machine learning approaches are beginning to address this gap by enabling predictive optimisation of activation conditions and adsorption performance from feedstock characterisation data [244].

### 6.5. Future Research Directions

The most consequential near-term opportunity for advancing the field is the integration of machine learning and artificial intelligence into carbon material design and process optimisation. ML tools can correlate synthesis parameters, including feedstock composition, carbonisation temperature, activation agent and ratio, and dwell time, with structural descriptors such as d-spacing, surface area, and pore volume. They reveal non-obvious relationships with functional performance metrics including specific capacitance, adsorption capacity, and catalytic yield, enabling rational exploration of parameter spaces that are intractable to exhaustive experimental screening [245,246]. Explainable AI approaches are particularly important for industrial adoption, where black-box models offer insufficient transparency for engineering decision-making, and their development requires interdisciplinary collaboration between materials scientists, data engineers, and process engineers [246]. The integration of Industry 4.0 concepts with ML models offers a pathway for scaling laboratory discoveries to continuous industrial production. These concepts include digital twins, real-time process monitoring, and automated feedback control, all of which help maintain the structural consistency that functional applications demand [247].

Standardisation of characterisation methodology represents an equally urgent priority. The lack of consensus on minimum reporting requirements, reference materials, and performance benchmarking criteria means datasets from different laboratories are not directly comparable. This limits the effectiveness of data-driven approaches and impedes meaningful cross-study evaluation of progress [204,246]. Internationally recognised standards for feedstock characterisation, processing condition reporting, structural characterisation protocols, and application-specific performance evaluation would create the data infrastructure necessary for both scientific progress and investor confidence in commercial deployment [246,248].

For energy storage applications specifically, systematic studies correlating the composition and structure of diverse biomass precursors with the hard carbon microstructure and sodium storage performance of the resulting anodes are needed. The current precursor-selection approach remains substantially empirical [204,246]. Scalable heteroatom doping strategies draw on the natural nitrogen, sulphur, and phosphorus content of biomass precursors while supplementing through controlled extrinsic doping. These require translation from laboratory synthesis to continuous industrial processes that can maintain doping configuration consistency at commercially relevant throughputs [246]. Circular economy implementation roadmaps that integrate techno-economic analysis, life cycle assessment, policy recommendations, and supply chain modelling are necessary for guiding the multi-stakeholder decisions required to move waste-derived carbon materials from research to commercial reality [246,249].

## 7. Conclusions

This review has demonstrated that the conversion of waste streams into functional carbon materials is not a single technology but a family of interconnected decisions about feedstock selection, processing route, structural engineering, and application targeting. Their outcomes are governed by the structure–property–function relationships examined throughout. The central argument is that waste-derived carbon materials are not inherently inferior substitutes for conventionally produced carbons but are demonstrably capable of matching or exceeding their performance when the processing pathway is rationally aligned with the structural requirements of the target application.

The feedstock categories reviewed in Section 2 establish that biomass, plastic waste, and industrial residues each offer distinct structural starting points whose compositional characteristics propagate through thermochemical processing into predictable carbon architectures. The processing pathways of Section 3, namely pyrolysis, hydrothermal carbonisation, activation, and microwave-assisted routes, provide the tools for shaping those architectures, but only when the mutual dependencies between feedstock chemistry and processing parameters are understood and exploited rather than ignored. The structural characteristics of Section 4 confirm that morphology, hierarchical porosity, surface chemistry, and crystallinity are not independent outcomes but co-determined features that must be considered together. Section 5 demonstrates quantitatively that performance in electrochemical energy storage, adsorption, catalysis, and high-temperature deployment follows directly from these structural features. This requires deliberate matching of pore size to target molecule, surface chemistry to interaction mechanism, and graphitisation degree to conductivity requirement.

The challenges of Section 6, namely feedstock variability, scale-up energy economics, emission management, and commercial feasibility, are real but not intractable. They are engineering and policy problems whose resolution depends on standardisation, continuous process development, life cycle thinking, and market frameworks that value the carbon sequestration and waste diversion benefits of these materials alongside their functional performance.

Three priorities emerge from the evidence in this review. KOH activation of lignocellulosic biomass consistently achieves BET surface areas above 2000 m^2^/g, yet the energy cost of activated carbon production remains a barrier to scale, and process integration with waste heat recovery is the most pressing engineering target. Surface chemistry rather than surface area alone determines adsorption selectivity, and heteroatom doping strategies must be matched to target contaminant chemistry. The absence of systematic multi-cycle and fixed-bed column data for waste-derived carbons is the principal gap between laboratory demonstration and industrial deployment.

## Figures and Tables

**Table 3:** Direct comparison of microwave-assisted and conventional thermal processing for selected carbon material production metrics.

Parameter	Conventional Method	Microwave-Assisted	Difference	Key References
Carbonisation time—rice husk catalyst	4 h	30 min	87% reduction	[61]
Sulphonation time—rice husk catalyst	12 h	20 min	97% reduction	[61]
BET surface area—biomass char	71 m^2^/g	138 m^2^/g	94% higher	[115]
Sulphur content—sulphonated catalyst	2.10%	4.91%	134% higher	[61]
BET surface area—catalyst surface area	37.01 m^2^/g	43.63 m^2^/g	18% higher	[61]
Processing time reduction—general biomass	Baseline	Up to 80% shorter	Up to 80%	[116]
BET surface area—activated carbon (maximum)	~920 m^2^/g	~2996 m^2^/g	~225% higher	[62,117]
CO_2_ uptake—ultra-microporous carbon	Baseline	5.3 mmol g^−1^ at 0 °C/1 bar	Among the highest reported	[63]
Graphitic character—Raman IG/Iall ratio	Lower	Higher	Superior structural ordering	[115]

**Table 4:** Key structural features of waste-derived carbon materials: characterisation methods, characteristic ranges, and functional performance implications.

Structural Feature	Primary Characterisation Technique	Characteristic Range	Key Functional Role	Performance Implication	Key References
Surface morphology	SEM	Feedstock-dependent; wood retains vascular channels; plastics form spheres	Determines pore geometry, transport pathways, and accessible surface	Wood-derived channels support fluid transport; spherical plastic chars require activation	[122,123,124]
Micropores (<2 nm)	BET N_2_ adsorption; CO_2_ adsorption (273K)	Surface area up to 3700 m^2^/g via KOH activation	Primary adsorption sites; electric double-layer charge storage	Dominant capacitance and CO_2_ uptake; high tortuosity limits rate performance	[125,126,127]
Mesopores (2–50 nm)	BET N_2_ adsorption; BJH analysis	Pore diameter 2–50 nm; facilitates access to micropores	Ion transport highways; diffusion pathways	Improves rate capability; prevents mass transfer limitation	[127,128,129]
Macropores (>50 nm)	Mercury intrusion; SEM	>50 nm; electrolyte reservoir	Ion buffering at electrode surface	Reduces diffusion distance; improves power density at high current	[128,130]
Pore connectivity	n-nonane pre-adsorption; SAXS; TEM 3D reconstruction	Quantified by LPPS; 10 nm achieved in best-performing HPC	Governs functional accessibility of surface area	Poor connectivity negates high BET surface area; 405 F/g at 200 A/g with optimised connectivity	[126,131,132]
Oxygen functional groups	FTIR; XPS	Higher at low pyrolysis temperature; reduced above 700 °C	Wettability; adsorption active sites; pseudocapacitance	Improved hydrophilicity; heavy metal complexation; faradaic charge storage	[133,134,135]
Nitrogen doping	XPS (pyridinic-N, pyrrolic-N, graphitic-N)	Typically 1–15 at.% achievable	Conductivity enhancement; catalytic active sites	Enhanced ORR activity; improved electrochemical performance; pseudocapacitive contribution	[136,137]
Sulphur/phosphorus doping	XPS; FTIR	Typically 0.5–5 at.% each	Interlayer expansion; electronic redistribution	Increased active sites; synergistic co-doping effects exceed single-element doping	[138,139,140]
Turbostratic structure	XRD (d_002_ = 3.42–3.50 Å); Raman (high ID/IG; symmetric 2D band)	d_002_ = 3.42–3.50 Å	Expanded interlayer spacing for Na^+^ accommodation; combined storage mechanisms	Up to 450 mAh/g in SIBs; stable over 250 cycles	[14,141,142]
Graphitic ordering	XRD (d_002_ approaching 3.35 Å); Raman (low ID/IG; split 2D band)	d_002_ approaching 3.35 Å	Electrical conductivity pathways; thermal and mechanical stability	Conductivity ~2.0 × 10^4^ S/cm in highly ordered films; reduced functional groups	[114,141,143]

BET = Brunauer–Emmett–Teller; BJH = Barrett–Joyner–Halenda; SAXS = small-angle X-ray scattering; HPC = hierarchical porous carbon; SIBs = sodium-ion batteries; LPPS = longest possible pore separation; ORR = oxygen reduction reaction.

**Table 5:** Selected quantitative structure–property–performance data for waste-derived carbon materials across principal application domains.

Application	Carbon Material	Key Structural Feature	Performance Metric	Key References
Supercapacitor	Heteroatom-doped hierarchical porous carbon	Hierarchical micro-meso-macropore; moderate defect density	612 F/g at 5 mV/s	[135]
Supercapacitor	Defect-engineered graphene blocks	Balanced defect density + pore structure; high compact density	52.2% capacitance retention at 20 A/g	[171]
Sodium-ion battery	Biomass-derived turbostratic hard carbon	d_002_ > 3.7 Å; microporous/mesoporous; vacancy defects	450/311 mAh/g (1st cycle); >90% retention over 200 cycles	[142,173]
CO_2_ capture	N-autodoped hierarchical porous carbon	Micropore-dominant; nitrogen defects for chemisorption	5.0 mmol/g; 230% increase over precursor	[183]
CO_2_ capture	P-doped porous carbon from lotus petiole	Pores < 2.02 nm dominant; P–C and P–O surface groups	2.51 mmol/g at 25 °C/1 bar; 90% uptake in 6 min	[182]
Organic dye adsorption	O–N–S doped hierarchical porous carbon (lotus leaf)	3601 m^2^/g; interconnected macro-meso-micropores; heteroatom wettability	9444 mg/g rhodamine B; >97% retention after 10 cycles	[185]
Organic dye adsorption	Hierarchical pore-broadened activated carbon	Mesopores dominant; pore size matched to Rhodamine B dimensions	881 mg/g rhodamine B vs. 374 mg/g microporous AC	[148]
Heavy metal adsorption	N-autodoped hierarchical porous carbon	Nitrogen defects for chemisorption; hierarchical pore for diffusion	434.8 mg/g Hg(II); equilibrium in 20 min	[183]
Phenol adsorption	N-doped hierarchical porous biochar (corn stalk)	High graphitic-N; microporous structure	Positive correlation: capacity scales with graphitic-N content	[181]
Biodiesel production	Sulfonated peanut shell carbon catalyst	–SO_3_H + –OH + –COOH synergistic acid sites	94.91% yield; 79.85% after 5 cycles	[193]
Biodiesel production	Superhydrophobic spherical activated carbon	High acid density (6.26 mmol/g); water-repelling surface	86.8% yield retained in 10th cycle	[195]
NO_x_ catalysis	MnO_x_/rice straw biochar	Surface functional groups as metal anchoring sites	84% NO_x_ removal at 50 °C	[189]
Electrochemical stability	Graphitised mesoporous carbon (CMK-3)	High graphitic ordering; mesopore preservation	Lower corrosion rate than Vulcan carbon black at 1.2 V vs. RHE	[198]
Thermal/oxidation resistance	Graphitic carbon coatings	Graphitisation level controls oxidation onset	Onset temperature 361–463 °C depending on graphitisation	[200]

## Data Availability

No new data were created or analyzed in this study. Data sharing is not applicable to this article.

## Abbreviations

The following abbreviations are used in this manuscript:

AD/AG	Defect-to-all-graphitic band intensity ratio (Raman)
BET	Brunauer–Emmett–Teller (surface area analysis)
BJH	Barrett–Joyner–Halenda (pore size distribution)
CFRP	Carbon fibre-reinforced polymer
CMK-3	Carbon mesostructure by KAIST (ordered mesoporous carbon)
DFT	Density functional theory
EU	European Union
FAME	Fatty acid methyl ester
FTIR	Fourier-transform infrared spectroscopy
H/C	Hydrogen-to-carbon atomic ratio
HDPE	High-density polyethylene
HPC	Hierarchical porous carbon
HTC	Hydrothermal carbonisation
ID/IG	Defect-to-graphitic band intensity ratio (Raman)
IG/Iall	Graphitic-to-total band intensity ratio (Raman)
IUPAC	International Union of Pure and Applied Chemistry
KOH	Potassium hydroxide
LDPE	Low-density polyethylene
LPPS	Largest pore-to-pore separation distance
LPSP	Lotus petiole-derived sulphur/phosphorus-co-doped carbon
ML	Machine learning
MSW	Municipal solid waste
NPV	Net present value
O/C	Oxygen-to-carbon atomic ratio
ORR	Oxygen reduction reaction
PCB	Printed circuit board
PE	Polyethylene
PET	Polyethylene terephthalate
PFAS	Per- and polyfluoroalkyl substances
PP	Polypropylene
PS	Polystyrene
Pt/C	Platinum on carbon (commercial electrocatalyst)
PVC	Polyvinyl chloride
RHE	Reversible hydrogen electrode
SAXS	Small-angle X-ray scattering
SEM	Scanning electron microscopy
TEM	Transmission electron microscopy
US	United States
VOC	Volatile organic compound
XPS	X-ray photoelectron spectroscopy
XRD	X-ray diffraction
ZnCl_2_	Zinc chloride
